# Implementation of a structured emergency nursing framework results in significant cost benefit

**DOI:** 10.1186/s12913-021-07326-y

**Published:** 2021-12-09

**Authors:** Kate Curtis, Prabhu Sivabalan, David S. Bedford, Julie Considine, Alfa D’Amato, Nada Shepherd, Margaret Fry, Belinda Munroe, Ramon Z. Shaban

**Affiliations:** 1 Susan Wakil School of Nursing, Faculty of Medicine and Health, University of Sydney, Office 169, RC Mills Building, Camperdown, NSW Australia; 2 Emergency Services, Illawarra Shoalhaven Local Health District, Wollongong Hospital, Crown St, Wollongong, NSW Australia; 3 Illawarra Health and Medical Research Institute, University of Wollongong, Wollongong, NSW Australia; 4 George Institute for Global Health, University of NSW, Kensington, Australia; 5 Faculty of Medicine and Health, University of Wollongong, Wollongong, NSW Australia; 6 Business School, University of Technology Sydney, Sydney, NSW Australia; 7 Performance Analysis for Transformation in Healthcare (PATH) Group, UTS Business School, Ultimo, NSW Australia; 8 Deakin University, School of Nursing and Midwifery, Geelong, NSW Australia; 9 Deakin University, Centre for Quality and Patient Safety Research in the Institute for Health Transformation, Geelong, Victoria Australia; 10 Centre for Quality and Patient Safety Research – Eastern Health Partnership, Box Hill, Victoria Australia; 11 System Financial Performance, NSW Ministry of Health, North Sydney, NSW Australia; 12 Illawarra Shoalhaven Local Health District, Warrawong, NSW Australia; 13 School of Nursing and Midwifery, University of Technology Sydney, Sydney, NSW Australia; 14 Research & Practice Development Unit, Northern Sydney Local Health District, St Leonards, Sydney, NSW Australia; 15 Marie Bashir Institute for Infectious Diseases and Biosecurity, University of Sydney, Westmead, NSW Australia; 16 Division of Infectious Diseases and Sexual Health, Westmead Hospital and the New South Wales Biocontainment Centre, Western Sydney Local Heath District and New South Wales Ministry of Health, Westmead, NSW Australia

**Keywords:** Emergency nursing, Emergency department, Framework, Cost benefit, Patient safety, patient deterioration

## Abstract

**Background:**

Patients are at risk of deterioration on discharge from an emergency department (ED) to a ward, particularly in the first 72 h. The implementation of a structured emergency nursing framework (HIRAID) in regional New South Wales (NSW), Australia, resulted in a 50% reduction of clinical deterioration related to emergency nursing care. To date the cost implications of this are unknown. The aim of this study was to determine any net financial benefits arising from the implementation of the HIRAID emergency nursing framework.

**Methods:**

This retrospective cohort study was conducted between March 2018 and February 2019 across two hospitals in regional NSW, Australia. Costs associated with the implementation of HIRAID at the study sites were calculated using an estimate of initial HIRAID implementation costs (AUD) ($492,917) and ongoing HIRAID implementation costs ($134,077). Equivalent savings per annum (i.e. in less patient deterioration) were calculated using projected estimates of ED admission and patient deterioration episodes via OLS regression with confidence intervals for incremental additional deterioration costs per episode used as the basis for scenario analysis.

**Results:**

The HIRAID-equivalent savings per annum exceed the costs of implementation under all scenarios (Conservative, Expected and Optimistic). The estimated preliminary savings to the study sites per annum was $1,914,252 with a payback period of 75 days. Conservative projections estimated a net benefit of $1,813,760 per annum by 2022–23. The state-wide projected equivalent savings benefits of HIRAID equalled $227,585,008 per annum, by 2022–23.

**Conclusions:**

The implementation of HIRAID reduced costs associated with resources consumed from patient deterioration episodes. The HIRAID-equivalent savings per annum to the hospital exceed the costs of implementation across a range of scenarios, and upscaling would result in significant patient and cost benefit.

## Background

In-hospital adverse events are associated with increased mortality, morbidity and treatment costs [1] and the incidence of them in emergency admission patients is more than double that of non-emergency patient admissions [2]. Australia’s 292 Emergency Departments (EDs) treated more than 8.2 million patients in 2019–20 [3]. ED patients generally have undiagnosed conditions and varying degrees of clinical urgency and severity [4].

When patients attend an ED, emergency nurses are the first health care provider to assess the patient and commence emergency care, so patient safety is contingent on their accurate assessment, interpretation of clinical data, intervention, early recognition of deterioration and escalation of care [5, 6]. Failure to recognise and respond to clinical deterioration during emergency care increases the incidence of high-mortality adverse events both during emergency care but also following the emergency care episode, irrespective of whether the patient is admitted to hospital or discharged [7, 8].

Clinical deterioration within 72 h of admission via the ED is an adverse event and can be associated with the care in ED [7, 8]. Patients admitted via the ED and who deteriorate on the ward during the early stage of their admission also have significantly higher in-hospital mortality [7, 9]. In our health district, the average (SD) treatment costs for patients who deteriorated within 72 h of hospital admission via the ED were tripled, irrespective of diagnosis, age or hospital length of stay (LOS) [10].

Health care organisations outlay significant funds for nurse education, education staff and online mandatory training, yet evaluations that yield information about the return on investment are scarce, particularly in relation to patient safety or health service outcomes [11]. The existing evidence base does not enable any empirical conclusions to be drawn about the economic value of continuing health professional development [12]. This study seeks to examine the outcome of investment in an intervention with a considerable nursing education component in relation to cost benefit.

**Fig. 1:**
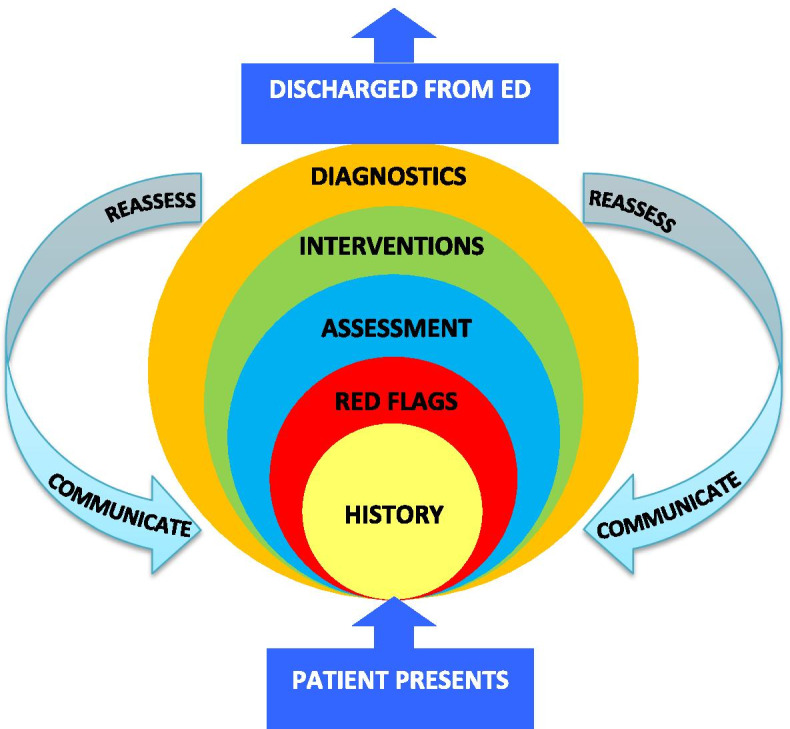
The HIRAID Emergency Nursing Framework.© Curtis, Munroe, Murphy, Strachan, Lewis & Buckley 2016, adapted from Curtis et al. 2009

Implementation of a nurse-led framework called HIRAID (History, Identify Red flags, Assessment, Interventions, Diagnostics, communication and reassessment) [13] was developed for emergency care delivery (Fig. 1) in our Local Health District. The findings of this study resulted in a 50% (27 to 13%) reduction of inpatient clinical deterioration associated with care in the ED as classified by the Human Factors Classification Framework for patient safety [14]. HIRAID is the only validated framework designed to enable emergency nurses to systematically assess and manage ED patients [15]. The cost of HIRAID implementation and any cost-benefit is unknown. The aim of this study was to determine the financial costs and payback period of implementing the HIRAID emergency nursing framework and any potential future net financial benefits as a result of decreased inpatient deterioration related to ED care.

## Methods

### Study design and setting

This retrospective cohort study was conducted between March 2018 and February 2019 across two hospitals in regional NSW, Australia. This study was approved by the site health and medical human research ethics committee (LNR/16/WGONG/249) and conducted per the approved protocol.

### Implementation of HIRAID

HIRAID was introduced to the EDs using a detailed implementation strategy, the development of which is reported elsewhere [16]. Modes of delivery selected to implement HIRAID included: (i) the development and compulsory completion of an eLearning module; (ii) attendance at a half day HIRAID workshop; (iii) integration of HIRAID into ED orientation programs and specialty training programs; (iv) mandated quarterly random audits of 10 episodes of initial nursing documentation at all sites; (v) introduction of cues within the workplace such as posters and reference cards; development and; (vi) mandated use of a documentation template based on the HIRAID assessment structure [16]. A template outlining the range of HIRAID implementation costs was generated. From this list of cost outlays, we determined the initial HIRAID implementation cost and ongoing annual implementation costs.

### Cost of patient deterioration during early stages of hospital admission

Similar to the majority of high income countries [17], Australia uses diagnostic related groups to calculate public hospital funding on an activity basis, specifically, Australian Refined Diagnosis Related Groups (AR-DRGs) [18]. AR-DRGs group patients with similar diagnoses requiring similar hospital services and is updated every 3 years along with the International Statistical Classification of Diseases and Related Health Problems, Australian Classification of Health Interventions and Australian Coding Standards classification (ICD-10-AM/ACHI/ACS). Episodes of admitted acute care are assigned with disease and intervention codes by health information managers or clinical coders. AR-DRGs are then assigned based on these codes and a number of other routinely collected variables including age, sex, mode of separation, length of stay, newborn admission weight and hours of mechanical ventilation [18].

Data on patients experiencing clinical deterioration within 72 h of admission were provided to the site costing unit. Clinical deterioration was defined as a cardiac arrest, unplanned intensive care unit admission or a rapid response call [19]. Staff can activate a rapid response call when they are concerned patient deterioration needs immediate medical review by the critical care team. The criteria for activation of a rapid response call are standardised across the NSW State health system [14]. The AR-DRGs (v8.0) of these patients were extracted, and a cost comparison between those who did and did not have a deterioration episode was recorded. The top 10 AR-DRGs were also compared. Treatment costs included direct, indirect and corporate overhead costs. When controlled for confounders (LOS, AR-DRG code and others), the average incremental cost of clinical deterioration in the first 72 h of hospital admission via the ED was $2591.14 (CI +/− $654.92) [19]. These costs were used as the basis for the analyses in this study. The currency presented is Australian dollar (AUD).

### Data analysis

Data were cleaned (validations and definitions) and collated for descriptive analysis. Data were analysed using Stata Version 14.2 (StataCorp, College Station, USA) to test if the deterioration and non-deterioration patient groups were equal in characteristics. T-tests or Mann Whitney U tests were used for the comparison of continuous variables. Chi-square tests were used for categorical variables. All statistical tests were conducted as two tailed, and a confidence level of 95% was used to determine if there was a significant association between the cohorts and variables of interest. For the cost analyses, there were two main calculations within the analysis: i) hospital level net benefit figure; and ii) payback period. The hospital level net benefit figure was calculated by offsetting the cost of savings per episode of clinical deterioration within 72 h of admission via the ED, against the initial and ongoing implementation costs per hospital from the HIRAID initiative. We used the confidence intervals from Curtis et al. (2021) [19] as the basis for an *optimistic* ($2591.14 + $654.92) and *conservative* case ($2591.14 - $654.92) for the value of savings per episode of deterioration, with the *expected* case being $2591.14 itself.

We then multiplied this value by the total expected patient deterioration in ED across NSW, estimated by taking the deterioration percentage (929 deterioration episodes/25,026 total ED admissions for our measured 352 common AR-DRG categories) in the HIRAID test sites [20]. This reflects a 3.7% deterioration proportion. That percentage was multiplied by the total projected ED admissions figure, initially obtained for 2018–19 using the NSW government health data portal [21], with 2% growth estimates conservatively estimated (the prior 7 years revealed an average growth rate higher than 2.5%). Multiplying these by the deterioration percentage gave us a deterioration estimate projection. The deterioration saving cost (conservative, expected and optimistic) were obtained by multiplying the relevant average cost of deterioration savings by the deterioration episode estimate, to reveal the state level projections. The hospital level savings projections were estimated by identifying a per hospital average of 465 deterioration encounters – obtained by dividing the 929 deterioration encounters noted in across both ED departments where the data were collected [20]. We did not factor in a nominal or real inflation rate on the rate of increase in pricing to be even more conservative with our savings estimates.

The variable formula calculations from the site level and state level are as shown below:

**Table:** 

**Step 1: Variable formula (site/hospital level):**	
Conservative	1936.22 savings per encounter * Det. encounter volume - Initial program investment = Net Savings in sample sites
Moderate	$2591.14 savings per encounter * Det. Encounter volume - Initial program investment = Net savings in sample sites
Optimistic	$3246.06 savings per encounter * Det. Encounter volume - Initial program investment = Net savings in sample sites
**Step 2: Variable formula (state level for all three scenarios)** Relevant ARDRG ED admissions estimate * Net savings per encounter = Net state level savings	
The variable definitions relating to the two formulas from Table 3 above, are explained below:	
**Step 1 formula (site level)**	
*Savings per encounter:* Extracted from Curtis, et al. (2021) with conservative, moderate and optimisitic savings estimated using the standard deviation estimates from the same data.	
*Deterioration encounter volume at the site level:* as extracted from the site empirics, per Curtis et al. (2021)	
Initial program investment - comprised of initial training, manual and staff time related costs associated to the establishment of the program.	
**Step 2 formula (for all three state scenarios, Conservative, Moderate and Optimistic)**	
*Relevant ARDRG ED admissions estimate:* (Site level relevant ARDRG admissions/site level total ED admissions) * state-wide ED admissions	
*Net savings per encounter:* Extracted from Curtis, et al. (2021) with conservative, moderate and optimistic savings estimated using the standard deviation estimates from the same data.	

The payback period estimates how quickly the setup costs of an initiative, in this case, the implementation of HIRAID is “paid-back” or covered by the equivalent value of its benefits generated (reduction in deterioration). By dividing the implementation cost per annum by the equivalent per annum savings estimate and multiplying the resulting number by 365 days, we generated a payback period – an assessment of how quickly the HIRAID investment “paid” for itself on an annual basis. The Payback period variable formula to express the above is as follows: Payback period = (Initial cost outlay/Total net savings) * 365 days.

## Results

The estimated initial HIRAID implementation cost in Year 1 was $492,917, and ongoing implementation costs $134,077 per annum (Table 1).

**Table 1:** HIRAID initial and ongoing implementation costs

	Initial outlay hours and line activity cost estimate								Ongoing outlay hours and line activity cost estimate						
Development and Production Activity	Hrs CNC	Hrs NE	Hrs SS	Hrs RN3	Hrs HSM2	Hrs CNE	Cash outlay	Cost by line activity	Hrs CNC	Hrs NE	Hrs SS	Hrs RN3	Hrs HSM2	Hrs CNE	Cost by line activity
eLearning module	40	4	16	4	4	4	0.00	$6486	20	4	4	2	2		$2713
Demo video for eLearning module	6	4	0	2	2	2	0	$1154	6	4	0	2	2		$1023
Revision of orientation manual	30	2	0	0	0	8	0	$3117	30	2	0	0	0	8	$3117
Curriculum development, train the trainers course. 58 senior staff over 8 days	260	64	0	512		24		$50,982	130	64	0	192		24	$50,982
RN 1 h training				220		24		$11,668				220		24	$11,668
Clinical champion nurse for each ED						896		$58,274						896	$58,274
Teaching manuals	8	8				8		$1756	8	8				8	$1756
Posters / Flipcards	2					8		$683	2					8	$683
Additional CNE time over implementation						564		$36,681							$ -
Promotional video	25							$2042	25						$2042
Documentation templates	2	1				1		$301	2	1				1	$301
Altering of policy	5							$408	5						$408
Audit tool	6							$490	6						$490
CNE, NUM time / meet	4	4						$618	4	4					$618
Evaluation							318,255	$318,255							$ -
Total cost								$492,917							$134,077

*CNC* Clinical nurse consultant, *NE* Nurse educator, *SS* Staff Specialist – Emergency Physician, *RN* Registered Nurse, *HSM* Health service manager, *CNE* Clinical Nurse Educator

Of the 25,062 patients included in the study 929 patients experienced an episode of deterioration within 72 h of admission via the ED (Table 2). Patients who deteriorated were significantly older (median 73.4 vs 67.5 years, *p* <  0.001) and had a longer median ED length of stay (9.0 vs 7.0 h, *p* <  0.001), LOS (10.48 vs 8.99 days, *p* <  0.001) than patients who did not deteriorate. For patients who had an ICU admission, patients who deteriorated within 72 h of admission via the ED had significantly longer ICU length of stay (3.74 vs 3.18 days, *p* <  0.001) than patients who did not deteriorate.

**Table 2:** Patient characteristics, emergency and hospital LOS by deterioration vs no deterioration event within 72 h of admission via ED

Variable	No deterioration within 72 h (***n*** = 24,133)	Deterioration within 72 h (***n*** = 929)	***p***-value
Age – Median (IQR)	67.5 (44.9–80.9)	73.4 (60.8–83.0)	< 0.001
Gender – n (%)			
Male	11,518 (47.7)	474 (51.0)	0.048
Female	12,615 (52.3)	455 (49.0)	
ED LOS (h) – Median (IQR)	7.0 (3.9–12.3)	9.0 (5.4–14.4)	< 0.001
Site – n (%)			
Site 1	18,488 (76.6)	656 (70.6)	< 0.001
Site 2	5645 (23.4)	273 (29.4)	
Time of presentation – n (%)			
Morning (07:00–15:00)	11,166 (46.3)	462 (49.7)	0.116
Afternoon (15:01–22:00)	8876 (36.8)	319 (34.3)	
Night (22:01–06:59)	4091 (17.0)	148 (15.9)	
Time of admission – n (%)			
Morning (07:00–15:00)	7683 (31.8)	282 (30.4)	0.465
Afternoon (15:01–22:00)	9611 (39.8)	368 (39.6)	
Night (22:01–06:59)	6839 (28.3)	279 (30.0)	
Average ED LOS (hours)	8.99 (6.51)	10.48 (6.65)	< 0.001
Average hospital LOS (days)	4.54 (5.81)	12.47 (12.41)	< 0.001
Average ICU LOS (days)(ICU cases only)	3.18 (4.15)	3.74 (4.82)	< 0.001

### Hospital net benefit

The hospital level net benefit figure in the first year of HIRAID implementation ranged from $1,305,831 (conservative) to $2,522,673 (optimistic). The expected ongoing annual hospital net benefit figure was $2,472,610. This was calculated by multiplying the cost of savings per episode by the 465 average savings encounters per hospital (929 deterioration savings encounters/2 sites), and subtracting initial and ongoing implementation costs per hospital from this amount, as identified from HIRAID site estimates ($492,917 initial, ongoing $134,077 per annum). Staff research costs dominated initial year costings. These would be far less in new hospital sites, but we conservatively chose to include them. We showed net savings under all scenarios, across all 5 years, even when implementation cost concerns were considered (Table 3).

**Table 3:** Analysis of savings and net benefits from prevention of clinical deterioration in ward patients during the early stages of emergency admission

Outcome	2018–19	2019–20	2020–21	2021–22	2022–23
ED admission growth rate	3.34%	2.00%	2.00%	2.00%	2.00%
Projected ED admissions (352 AR-DRG)	2,189,030.929	2,232,811.547	2,277,467.778	2,323,017.134	2,369,477.477
Statewide deterioration episode projection	81,143	82,766	84,421	86,110	87,832
Expected equivalent savings (**state level estimates**)					
Conservative ($1936.22 per episode)	$157,110,699	$160,253,185	$163,457,629	$166,727,904	$170,062,075
Expected ($2591.14 per episode)	$210,252,873	$214,458,293	$218,746,630	$223,123,065	$227,585,008
Optimistic ($3246.06 per episode)	$263,395,047	$268,663,402	$274,035,631	$279,518,227	$285,107,942
Net benefit - **hospital level estimates**: expected equivalent savings less HIRAID implementation costs:					
*Conservative ($1936.22 per episode)*	$1,798,748	$1,835,537	$1,872,325	$ 1,909,113	$ 1,947,837
Implementation costs (initial and ongoing)	-$492,917	-$134,077	-$ 134,077	-$ 134,077	-$ 134,077
*Net savings*	$ 1,305,831	$1,701,460	$1,738,248	$ 1,775,036	$ 1,813,760
Payback period days: (Inv. Outlay/Det. Savings) * 365 days	*100.02*	*26.66*	*26.14*	*25.63*	*25.12*
*Expected ($2591.14 per episode)*	$2,407,169	$2,456,401	$2,505,632	$ 2,554,864	$ 2,606,687
Implementation costs (initial and ongoing)	-$492,917	-$134,077	-$134,077	-$134,077	-$ 134,077
*Net savings*	$1,914,252	$2,322,324	$2,371,555	$420,787	$2,472,610
Payback period days: (Inv. Outlay/Det. Savings) * 365 days	*74.74*	*19.92*	*19.53*	*19.15*	*18.77*
*Optimistic ($3246.06 per episode)*	$3,015,590	$3,077,265	$3,138,940	$3,200,615	$3,265,536
Implementation costs (initial and ongoing)	-$492,917	-$134,077	-$134,077	-$134,077	-$134,077
*Net savings*	$2,522,673	$2,943,188	$ 3,004,863	$3,066,538	$3,131,459
Payback period days: (Inv. Outlay/Det. Savings) * 365 days	*59.66*	*15.90*	*15.59*	*15.29*	*14.99*

* Hospital deterioration episode encounter projection is 465, a whole number average per hospital deterioration from the 929 deteriorations observed over the two sites (Table 1)

### Hospital payback period

For return of investment for the initial implementation of HIRAID, the longest hospital payback period was 100 days (conservative scenario, 2018–19) and the quickest (shortest) payback period was 60 days (optimistic scenario, 2018–19). For ongoing investment and sustained implementation of HIRAID, the longest hospital payback period was 26 days (conservative scenario, 2018–19) and the quickest (shortest) payback period was 15 days (optimistic scenario, 2022–23). These are all well under a year, meaning the health service re-obtains their investment via the opportunity cost saving of deterioration avoidance.

### NSW state-wide projected savings and payback period

State-wide HIRAID implementation projected savings were calculated at $227,585,008 per annum for hospitals with an ED. The total projected ED admissions figure with 2% growth estimates conservatively estimated yielded the State-wide ED admissions shown in Table 3. The spectrum of cost savings possibilities presented statistically relates to 90% of possible outcomes (optimistic being in the top 5 percentile, conservative being in the bottom 5 percentile). For each scenario, the statistically derived equivalent savings per episode was multiplied against the number of deteriorations to calculate a savings figure.

## Discussion

This study determined the initial investment required for and net financial benefits arising from the implementation of the HIRAID emergency nursing framework at a hospital and State-wide level. The implementation of HIRAID resulted in an estimated cost benefit of $1,914,252 to the study sites with a 75-day payback period. State-wide implementation of HIRAID could save NSW public hospitals $277million per year as a result of decreased inpatient deterioration. These findings speak strongly in favour of the benefit of HIRAID economically, in addition to its clear patient level benefits.

Nurses are by far the largest proportion of the professional health workforce. Emergency care presentations are increasing exponentially around the world and quality nursing care is fundamental to patient safety [4]. The World Health Assembly (WHA) 2019 draft resolution recommended emergency care training through speciality training programmes [22]. This study demonstrates that such an initiative could potential result in financial savings to the implementing health care system.

The application of HIRAID is not dependent on context, clinical skill level or resources. The operationalisation of HIRAID as a basic assessment process, and foundation for nurse-initiated care protocols can be readily adapted for implementation in other international jurisdictions [23]. HIRAID train-the-trainer courses have been delivered in Sri Lanka, Fiji, Nepal and Colombia. However, HIRAID has only been tested in Australia [14, 16]. HIRAID requires formal consultation with emergency nurses internationally [23].

The implementation of HIRAID required an initial investment, particularly to conduct education and training. This investment was rapidly offset in all three projected scenarios to 2022–23 All health professional education and introduction of interventions within ED come with significant costs, which we have explicitly described in this study. The importance of planning and investment in implementation cannot be understated. There are many instances of less than adequate implementation results in the ED setting where clinician behaviour change is difficult to achieve [24–27]. Successful implementation needs appropriate funding, planning and strategies that address the complexity and micro-politics embedded within all health care systems. Implementation strategies need to support individual practitioners, managers, and understand the context as well as receive strong organisational support and patronage which is influential to normalising a new practice among staff [28]. An evidence informed and context specific implementation strategy is essential to sustained, reliable and high uptake [16, 29]. While education and training is accompanied by associated cost, this study has shown that HIRAID can lead to significant cost benefits and pay back for an organisation. We recommend the employment of a HIRAID nurse for 18 months to implement, embed and monitor uptake that is tailored for each ED context.

Prevention and early identification of patient deterioration improves outcomes, quality of life and lessens the intervention required to stabilise patients whose condition deteriorates unexpectedly in acute health service organisations [2]. Several health service wide interventions have been implemented to address the multiple complex organisational and workforce factors that contribute to patient deterioration [30]. Nonetheless, avoidable patient deterioration rates continue as a result of failure to recognise and rescue. Across the literature recognizing patient deterioration comprises four key areas: (1) assessing the patient; (2) knowing the patient; (3) education and (4) environmental factors [31]. The HIRAID framework [13], and accompanying implementation strategy [29] encompasses these areas ensuring emergency nursing staff have the capability, capacity and opportunity to apply HIRAID in their clinical practice.

Future research should include the evaluation of other benefits of improved emergency nursing care, nurse sensitive adverse events and patient deterioration. For example, the reduced LOS that is generated through reduced patient deterioration [14] and nurse sensitive adverse events [32] may create additional inpatient capacity. This in turn could improve associated key performance indicators such as non-compliance with emergency treatment performance (ETP), which is an independent predictor of all cause 30-day mortality for patients presenting to, and admitted via ED [33].

There are limitations to this study. Although all care was taken in the identification and assessment of patient deterioration events and a standardised process used, it is possible some were missed. Although we examined ED care related causal factors to the deterioration event [14], we did not collect information on potential ward based factors for the event, such as staffing levels. This study was conducted in one health district, and despite the incidence of patient deterioration and adverse events in all hospitals, the types of incidences may differ, reducing the applicability to other hospitals. Cost estimates are likely to differ between countries, institutions and populations, thus potentially limiting the generalisability of this and all cost-effectiveness studies in health education [34].

## Conclusions

The implementation of a structured emergency nursing framework resulted in substantial cost benefit with payback of investment within a year. The State-wide implementation of HIRAID could save $227million per year. Initial investment in a dedicated senior implementation nurse is crucial for successful and sustained uptake.

## Data Availability

The data that support the findings of this study are available from the University of Wollongong HREC but restrictions apply to the availability of these data, which were used under license for the current study, and so are not publicly available. Data are however available from the authors upon reasonable request and with permission of the University of Wollongong HREC.
